# DNA-PKcs inhibitor AZD7648 reveals sgRNA cross-contaminants and enhanced sensitivity of genome engineering off-target activity in HSPCs

**DOI:** 10.1093/nar/gkag318

**Published:** 2026-04-20

**Authors:** Nathan White, Yi-Ting Hu, John Alexander Chalk, Gavin Kurgan, Asma Naseem, Ellen Schmaljohn, Morgan Sturgeon, Alessia Cavazza, Adrian James Thrasher, Giandomenico Turchiano

**Affiliations:** Infection, Immunity, and Inflammation Teaching and Research Department, Great Ormond Street Institute of Child Health, University College London, London, WC1N 1DZ, United Kingdom; Infection, Immunity, and Inflammation Teaching and Research Department, Great Ormond Street Institute of Child Health, University College London, London, WC1N 1DZ, United Kingdom; Infection, Immunity, and Inflammation Teaching and Research Department, Great Ormond Street Institute of Child Health, University College London, London, WC1N 1DZ, United Kingdom; Clinical Pharmacology and Safety Sciences, R&D, AstraZeneca, Cambridge, CB2 0AA, United Kingdom; Integrated DNA Technologies, Coralville, IA 52241, United States; Infection, Immunity, and Inflammation Teaching and Research Department, Great Ormond Street Institute of Child Health, University College London, London, WC1N 1DZ, United Kingdom; Integrated DNA Technologies, Coralville, IA 52241, United States; Integrated DNA Technologies, Coralville, IA 52241, United States; Infection, Immunity, and Inflammation Teaching and Research Department, Great Ormond Street Institute of Child Health, University College London, London, WC1N 1DZ, United Kingdom; Infection, Immunity, and Inflammation Teaching and Research Department, Great Ormond Street Institute of Child Health, University College London, London, WC1N 1DZ, United Kingdom; Infection, Immunity, and Inflammation Teaching and Research Department, Great Ormond Street Institute of Child Health, University College London, London, WC1N 1DZ, United Kingdom; Clinical Pharmacology and Safety Sciences, R&D, AstraZeneca, Cambridge, CB2 0AA, United Kingdom

## Abstract

Therapeutic gene editing with designer nucleases can be compromised by undesired repair outcomes. DNA repair inhibitors are used to bias DSB repair toward HDR, but their impact on larger structural rearrangements, including large deletions and translocations, remains unclear. We quantify the mutational burden associated with end-joining inhibitor compounds. With a highly precise Cas9 nuclease, repair inhibition yields modest increases in aberrations, whereas promiscuous single guide RNAs (sgRNAs) amplify aberrant outcomes by orders of magnitude. Donor templates mitigate mutational burden at on-target sites, and in rare cases donor sequences bridge translocations between on- and off-target loci. Because DNA-PKcs inhibition does not itself induce instability over short intervals but increases the likelihood of capturing chromosomal aberrations postediting, we leveraged this to enhance assay performance. Compared to CAST-Seq, high-resolution CAST-Seq achieved a median ~12-fold increase in detected aberrations and, in this higher-sensitivity context, revealed unintended, target-specific sgRNA contaminants in GMP-like batches, underscoring direct genotoxicity risk and the need for stricter guide purity controls. A modified, translocation-quantitative rhAmpSeq reports all translocation combinations between two loci, enabling robust off-target validation beyond indel-only readouts. Finally, we evaluate AZD7648, finding limited aberration increases with precise nucleases and reconciling reports of extensive large deletions by quantifying assay- and design-dependent biases.

## Graphical Abstract

**Graphical Abstract:**
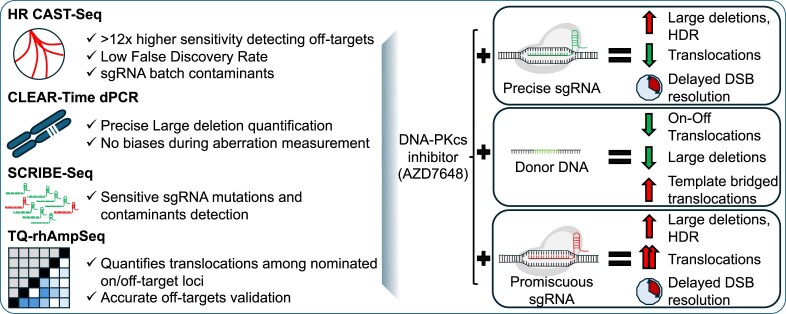

## Introduction

Precise genetic sequence insertions can be facilitated with programmable endonucleases such as clustered, regularly interspaced, short palindromic repeats (CRISPR)–associated protein 9 (Cas9) and an exogeneous donor template [1, 2]. However, the efficiency of template-mediated homology-directed repair (HDR), whether through AAV transduction, single-strand oligodeoxynucleotide (ssODN) delivery, or other methods, is contingent on cell cycle progression to activate the specific pathway [3, 4]. Targeted integration enhancers (TIEs) have been employed to mitigate these limitations by inhibiting NHEJ components (Ku70/80 [5], TP53BP1 [6], DNA ligase IV [7]), overexpressing HDR-related proteins (e.g. RAD51 [8]), or synchronizing the cell cycle [9], but many such strategies are non-selective, incompatible with clinical translation or poorly effective [10, 11]

Competing repair pathways generate undesired outcomes, including indels, large deletions, translocations, and other structural aberrations, that erode efficacy and raise safety concerns [12, 13]. Gene fusions formed by nuclease-induced translocations have been observed in edited cells [14], and even co-delivery of two non-double-strand breaks (DSBs)-inducing base editors can induce translocations in human HSPCs [15], underscoring the potential for unintended chromosomal rearrangements. Off-target Cas9 cleavage further contributes to genotoxic by-products [16–18], yet the apparent efficiency at off-target loci, largely governed by single guide (sgRNA) mismatch tolerance [19], is often masked because cells repair many DSBs precisely via NHEJ or HDR [20, 21]. This not only poses a challenge when attempting to study such activity but also hinders the identification of rare and potentially detrimental aberrations.

The use of TIEs to simultaneously inhibit NHEJ and microhomology-mediated end joining (MMEJ) pathways using selective and potent DNA-PKcs (AZD7648) and Polθ (ART558) inhibitors, respectively, has emerged as an effective means to enhance template-mediated HDR while apparently minimizing unintended aberrations [22–25]. Although the increased efficiency of intended repair outcomes using DSB repair-inhibitors is clear, their effects regarding off-target activity and the associated induction of larger structural aberrations need deeper investigation [21, 26, 27].

Recent publications have clarified the impact of biases affecting certain techniques such as long-read sequencing, T7E1, and other polymerase chain reaction (PCR)-based assays [21, 28, 29] potentially misclassifying up to 90% of studied loci [21] that are affected by DSB, chromosomal aberrations, and large deletions.

Here, we assess designer nucleases with and without TIEs while explicitly accounting for these assay biases, providing a clearer view of the genomic consequences of modulating DNA-repair pathways in therapeutic editing. We introduce a set of complementary, high-sensitivity tools: high-resolution chromosomal aberrations analysis by single targeted linker-mediated PCR sequencing (HR-CAST-Seq), translocation-quantitative RNase H-dependent Amplicon Sequencing (TQ-rhAmpSeq), highly multiplexed CLEAR-time dPCR for translocations, and SCRIBE-Seq to detect and quantify single guide RNA (sgRNA) contamination. We further clarify the risk profile of TIEs by contrasting a promiscuous sgRNA with a highly stringent design, showing that precise nuclease selection yields only modest number of gross chromosomal aberrations, while revealing hidden liabilities when guides are less specific.

## Materials and methods

### Cell culture

CD34^+^ HSPCs were isolated by positive selection from conditioned peripheral blood from healthy male donors using the human CD34 MicroBead Kit (Miltenyi Bioscience) following the manufacturer’s instructions. Viability and purity were assessed with flow cytometry. Cells were cultured at 10^6^ cells/ml at 37°C, 5% CO_2_ in StemSpan SFEM Haematopoietic Cell Culture Medium (Stemcell Technologies), supplemented with Recombinant Human TPO (100 ng/ml; Peprotech), Recombinant Human SCF (100 ng/ml; Peprotech), Recombinant Human Flt3-Ligand (100 ng/ml; Peprotech), Recombinant Human IL-3 (100 ng/ml; Peprotech), and penicillin–streptomycin (1%; Gibco). Cell cultures were passaged every 2–3 days. See Supplementary Tables S1–S5 for additional materials information.

### Genome editing

SpCas9 (30 pmol; Integrated DNA Technologies) was complexed with an sgRNA (225 pmol; Synthego, CRISPRevolution sgRNA EZ Kit) to form a ribonucleoprotein (RNP) complex for 10 min at room temperature. See Supplementary Table S2 for the full list of sgRNA sequences used. 1.5 × 10^6^ HSPCs were resuspended in 20 µl P3 nucleofector solution with the complexed RNPs and electroporated in 16-well Nucleocuvette Strips using the 4D-Nucleofector system (Lonza; program CA-137). For experiments using ssODNs, 10 μM of ssODN was used in the final transfection reaction and co-electroporated with complexed RNPs. Electroporated HSPCs without complexed RNPs served as unedited controls.

### Small molecule compounds and treatment

HSPCs were cultured in medium containing DNA-PKcs inhibitor AZD7648 (Cambridge bioscience; HY-111783-5mg) (1μM) and/or POLQ inhibitor ART558 (3μM) (Cambridge bioscience; HY-141520-5mg) from 0 to 24 h post-editing, followed by a cell wash and culture in control medium.

### Genomic DNA extractions

DNA extractions were carried out using Monarch Genomic DNA Purification Kit (New England Biolabs; NEB) following the manufacture instructions. DNA concentration was quantified using a Nanodrop™ OneC UV-Vis Spectrophotometer.

### Indel quantification

DNA extracted from cells 3 days post-nucleofection were PCR-amplified with sequencing primers (see Supplementary Table S1 for a complete list of primers used) and purified using Monarch PCR and DNA Clean-up Kit (NEB). For Sanger sequencing, samples were processed using the Eurofins Genomics TubeSeq NightXpress service with 2 µl forward primer pre-mixed with 15 µl PCR-purified amplicons (10 ng/µl). Indel frequency assessment was performed using inference of CRISPR editing (ICE) analysis (Synthego Performance Analysis, ICE Analysis, 2019, v3.0. Synthego). For next generation sequencing (NGS), regions of interest were PCR-amplified and purified using a PCR and DNA Cleanup Kit (NEB, #T1130). To add Illumina adaptors to the amplicons, amplicons were processed using the NEBNext^®^ Ultra™ II DNA Library Prep Kit for Illumina (NEB, #E7645S), and indexed using NEBNext^®^ Multiplex Oligos for Illumina^®^ (96 Unique Dual Index Primer Pairs) (NEB, #E6440S) following the manufacturer instructions. Indexed amplicons were then purified using AMPure XP Bead-Based Reagent (BECKMAN COULTER) at 0.9× concentration following the manufacturer instructions. The amplicon libraries were quantified using droplet digital PCR with the ‘ddPCR Library Quantification Kit for Illumina TruSeq’ (Bio-Rad) following the manufacturer instructions.

### Next generation sequencing

NGS based techniques such as amplicon sequencing, TQ-rhAmpSeq and HR CAST-Seq produced libraries that were barcoded with indexed primers to allow for multiplexed run. The indexed libraries were denatured with NaOH and diluted to 10–20 pM, supplemented with 20% denatured PhiX Control v3 (Illumina) and sequenced using MiSeq (Illumina) with reagent Kit v3 (600 cycles) or reagent Kit v2 (300 cycles).

### Large deletion sequencing

Amplicons ~5000 bp length were generated by PCR reaction with primers flanking ~2500 bp of cleavage site and purified using AMPure XP Bead-Based Reagent (BECKMAN COULTER). Three hundred nanograms of amplicons were fragmented, end-repaired, and dA-tailed using NEBNext Ultra II FS DNA Library Prep Kit (NEB). The fragments were then ligated with adapters from the DNA Library Prep Kit, following the manufacturer’s guidelines. Libraries were purified with AMPure XP Bead-Based Reagent and barcoded using NEBNext Multiplex Oligos for Illumina (Dual Index Primer Pairs, NEB) for NGS. Finally, double-size selected purification was performed with AMPure XP Bead-Based Reagent to obtain 200–600-bp-length amplicons. Briefly, the purified amplicons were incubated with 0.5 times the total volume of magnetic beads for 5 min, magnetically separated and the harvested supernatant incubated with 0.2 times the volume of beads, again for 5 min. The supernatant was removed, and the purified amplicons remained, followed by two 80% ethanol washes, and eluted in TE buffer. The amplicon libraries were then quantified using droplet digital PCR with the ddPCR Library Quantification Kit for Illumina TruSeq (Bio-Rad). The denatured library (10–20 pM) was supplemented with 20% PhiX Control v3 (Illumina) and sequenced using MiSeq (Illumina) with reagent Kit v3 (600 cycles).

### rhAmpSeq and TQ-rhAmpSeq

For rhAmpSeq, 50 ng DNA was mixed with 4× rhAmpSeq CRISPR Library Mix 1 (IDT) and 10× rhAmp PCR Panel forward/reverse primer pool (with adapters) followed by an initial PCR (1× cycle at 95°C for 10 min, 14× cycles of 95°C for 15 s and 61°C for 8 min, 1× cycle at 99°C for 15 min). The initial PCR reaction was diluted 1 in 20, mixed with 4× rhAmpSeq CRISPR Library Mix 2 (IDT) and i5/i7 indexing primers (NEB), and subjected to a second round of PCR (1× cycle at 95°C for 3 min, 24× cycles of 95°C for 15 s and 60°C for 30 s, 72°C for 30 s, 1× cycle at 72°C for 1 min). Amplicons were then purified using AMPure XP Bead-Based Reagent (BECKMAN COULTER). For TQ-rhAmpSeq, the first PCR was performed with the modified rhAmpSeq primers without the extra adapter sequences. The purified PCR amplicons were then end repaired, A-tailed, ligated to an adapter, and purified following the protocol in NEBNext^®^ Ultra™ II DNA Library Prep Kit for Illumina^®^ and NEBNext^®^ Multiplex Oligos for Illumina^®^.

### sgRNA sequencing

Complementary DNA (cDNA) of the sgRNAs were synthesized by mixing 200 ng sgRNA with 2 mM dNTP mix (Thermo Fisher), and 1 μM sgRNA scaffold specific primer (Merck) and incubated at 70°C for 5 min. A reverse transcription mixture consisting of 1× Template-Switching RT Buffer (NEB), Template-Switching RT Enzyme Mix (NEB), and 3.75 μM adapter-linked template-switching oligo (TSO) was then immediately added to the primer annealing mixture and incubated at 42°C for 90 min and 85°C for 5 min. sgRNA cDNA was diluted 1:10 and subjected to a nested PCR using 0.2 μM adapter-linked primer upstream of the original RT scaffold specific primer and Q5 Hot-start HF 2× master mix (NEB). Amplicons were then purified using AMPure XP Bead-Based Reagent (BECKMAN COULTER) and barcoded following the 7 cycle PCR with NEBNext^®^ Multiplex Oligos for Illumina^®^.

### Droplet digital PCR

CLEAR-time dPCR was performed as described previously [21]. Briefly, edge, flanking, targeted integration, and relative size matched control assays (Supplementary Table S1) were performed in triplicate for each combination, DNA (25 ng per assay) was mixed with 2× digital PCR supermix for probes (No dUTP) (Bio-Rad), vortexed, and distributed with equal volumes into a ddPCR 96-Well Plates (Bio-Rad). Individual assay mixtures composed of primers (0.8 µM) and probes (0.25 µM) were then added to the ddPCR 96-well plates containing the DNA/supermix mixture. For the highly multiplexed translocation dPCR, a master mix consisting of 20 off-target forward or reverse primers, a common on-target reverse or forward primer, a pair of reference primers (0.8 μM), a common on-target probe and a reference probe (0.25 μM) were mixed together with the DNA/supermix mixture. All probes used in this study were PrimeTime qPCR probes (IDT) with the 5′ end fluorescent dye modification (6-carboxyfluorescein ‘FAM’ or hexachlorofluorescein ‘HEX’), 3′ end quencher (Iowa Black^®^ FQ ‘IBFQ’), and “ZEN” internal quencher. All probes were designed have a melting temperature 5°C–9°C greater than the primers. Droplets were generated using Automated Droplet Generation Oil for Probes (Bio-Rad) and DG32™ Automated Droplet Generator Cartridges with the automatic droplet generator (Bio-Rad AutoDG), and the plate sealed with a PCR Plate Heat Seal (Bio-Rad) and PX1™ PCR Plate Sealer (Bio-Rad). PCR was performed using the C1000 Touch™ Thermal Cycler with 96-Deep Well Reaction (Bio-Rad) and droplets read using a QX600 Droplet Reader (Bio-Rad). Droplet thresholds were manually set using QX Manager Software (Bio-Rad, Version 2.3) and processed in Excel (Microsoft). See Supplementary Table S1 for the full list of primer and probes used and Supplementary Table S4 for all PCR cycling conditions.

### CAST-Seq

CAST-Seq was performed as previously [17]. We randomly fragmented 500 ng of genomic DNA using the NEBNext Ultra II FS DNA Library Prep Kit (NEB) to achieve an average size of 350 bp. Linker oligos were ligated to both ends of fragments, followed by an initial PCR utilizing an on-target bait primer, linker prey primer, and decoy primer to prevent amplification of on-target sequences. A second PCR was performed with nested primers and adapters to the amplified sequences. Samples were barcoded using NEBNext Multiplex Oligos for Illumina for NGS in a third PCR reaction, followed by PCR purification using AMPure XP Bead-Based Reagent (BECKMAN COULTER). Individual barcoded samples and the final pooled library were quantified by droplet digital PCR. The denatured library (10–20 pM) was sequenced using MiSeq (Illumina) with reagent Kit v3 (600 cycles) and supplemented with 20% PhiX Control v3 (Illumina). See Supplementary Table S1 for a complete list of primers. Interchromosomal template-bridged translocations (ITTs) characterization was derived by modification in the CAST-Seq positive selection parameters by incorporating donor specific sequences, homology region, and on-target specific sequences, *k*mer at 35 bases.

### Bioinformatic analysis 

CAST-Seq data were analysed as previously described [17]. Additionally, replicates across conditions were analysed simultaneously using the CAST-Seq analysis pipeline to minimize the exclusion of rare translocation events. To further ensure robust identification (in addition to the statistical filtering implemented within the CAST-Seq pipeline), a minimum of seven reads found independently in at least two replicates were required to be classified as ‘true’. Unedited HSPCs served as a control for statistical filtering of true events and for the identification of alignment biases and physiological non-nuclease included DSBs. ‘Natural break sites’ (NBS) were defined as translocations with no homology to the on-target site, whereas on-target-mediated translocations (OMT) and homology-mediated translocations (HMT) were classified depending on sequence homology >25 bases with the on-target locus or within a 5-kb region, respectively.

CAST-Seq data on Fig. 2a and Supplementary Fig. S2e were analysed utilizing 8 samples with a replicate each (16 datasets + 2 untreated), to increase sensitivity of the method. Same genomic DNAs and analysis was performed placing the bait on the most common chromosome 13 off-target. CCR5 CAST-Seq data on Supplementary Fig. S2a were derived by a different donor in respect to the one on Fig. 2a, and analysed grouping by extraction days: 4 samples from day 3 with their own replicates (8 datasets + 2datasets for UT); day 14 with 4 samples (8 datasets). WAS CAST-Seq data in Fig. 5 were performed utilizing 4 samples + 1 UT with 2 replicates each combining a total of 8 datasets. EMX1 CAST-Seq data were derived by 2 samples + 1 UT with 2 replicates each.

Large deletion sequencing and SCRIBE-Seq data were analysed using bespoke Galaxy workflows (version 24.0.rc1). For large deletion sequencing analysis, paired-end read FASTQ files were initially processed with trimmomatic [30] and aligned to the human genome (hg38) using Bowtie2 [31]. Sequencing depth at each base was calculated with Samtools [32], normalized against the base with the highest coverage, and plotted using GraphPad PRISM (version 8.0.1). For SCRIBE-Seq analysis, FASTQ files were initially processed using PEAR [33]. Reads were then filtered for the scaffold sequence immediately downstream of the spacer sequence and clipped to just the spacer sequence. This was then aligned to the expected sgRNA sequence (for scoring) and also against the human genome (hg38 canonical).

### Translocation analysis on rhAmpSeq/TQ-rhAmpSeq

To quantify translocations from editing, a custom bioinformatic pipeline using hypergeometric test and methodology similar to previously published tools CRISPECTOR [34] was utilized with the following variations. Expected primers were identified in reads using fg-idprimer (https://github.com/fulcrumgenomics/fg-idprimer; -k = 6, -K = 8, -S = 5, --max-mismatch-rate = 0.07). Following this, treatment/control pairs had their counts paired and primer count frequencies subjected to a one-tailed hypergeometric test with Benjamini–Hochberg correction (statsmodel v0.15.0; default settings) to calculate an adjusted *P*-value (p^adj^). Unexpected primer pairs with p^adj^ < .01 were classified as a translocation and quantified using this method if (i) the estimated translocation frequency exceeds 0.01%, (ii) the background frequency of the event was quantified to be normal (<1% frequency in control), and (iii) the site has at least 100× coverage of both amplicons used for calling the event. A classified translocation had the translocation frequency (P) calculated using the following equation: ${{P}_t} = \frac{{\ {{n}_t}}}{{({{f}_{\textit{total}}} + {{r}_{\textit{total}}}) \div 2}}.$

Where ‘*n*’ is equal to the count of the unexpected primer pair of interest, ‘*t*’ is the significant translocation being interpreted, ‘*f*’ is the total count of the shared forward primer events excluding the count participating in the ‘*n*’ translocation event, and ‘*r*’ is the total count of shared reverse primer events excluding the count participating in the ‘*n*’ translocation event. The translocation frequency is then adjusted by the background level frequency in the control by subtracting any translocation frequency observed in the control sample from the treatment frequency. Total translocation burden (*B*) was calculated using the following equation:


\begin{eqnarray*}
B = 1 - \ \mathop \prod \limits_t^{{{t}_n}} \left( {1 - {{P}_t}} \right),
\end{eqnarray*}

where ‘*t*’ is equal to a significant translocation and ‘*t*_n_’ is equal to the last significant translocation of all translocations. All translocations for the purposes of this equation are assumed to be occurring independently.

### Editing analysis on rhAmpSeq/TQ-rhAmpSeq

Demultiplexed FASTQs were analysed using CRISPAltRations [28] using the default analysis window size for Cas9 (8 bp) to identify CRISPR editing events. Significant off-targets were determined by binning counts of indels identified by location relative to the cut site and using a hypergeometric test with Benjamini–Hochberg correction (statsmodel v0.15.0; default settings) to create an adjusted *P*-value per bin per off-target (*P* < .05). Off-targets were classified as edited if (i) the comparison of treatment/control samples at the site had a bin with a significant *P*-value (*P* < .05), (ii) sufficient read coverage for the site (>100×) was obtained in all samples, (iii) significant edits occurred in a bin at or adjacent to the cut site after optimal alignment, (iv) the classified cumulative significant edits exceeded 0.01%, and (v) an average coverage frequency of at least 5× the ascribed cumulative frequency was observed (e.g. for 0.1% editing, at least 5000× coverage). For determining blunt integration rates of donor oligos, quantified insertions >10 bp in size from the CRISPAltRations output were aligned to the expected donor oligo sequence using a glocal biopython implementation of the Needleman–Wunsch aligner. Alignments with an alignment score greater than 50 were quantified as a tag integration event, and events were considered imperfect if any base was mutated or missing from the expected DNA sequence alignment.

### Statistics and reproducibility

All digital PCR experiments were performed as three technical replicates (or as otherwise stated in the text). Statistical analysis and graphical illustrations were performed using Prism [GraphPad, version 8.0.1 (255)]. Statistical significance was defined when *P*-values were <.05, all exact *P*-values are addressed within figure legends where possible. All statistical tests performed were two-tailed unless stated otherwise. No data were excluded from the analyses.

### Ethics statement

Informed written consent was obtained from healthy donors for the use of human CD34+ haematopoietic stem and progenitor cells (HSPCs), in accordance with the Declaration of Helsinki. Ethical approval was granted by the Great Ormond Street Hospital for Children NHS Foundation Trust and the Institute of Child Health Research Ethics Committee (08/H0713/87).

AstraZeneca has a governance framework and processes in place to ensure that commercial sources have appropriate patient consent and ethical approval in place for the collection of samples for research purposes, including use by for-profit companies.

## Results

### DSB repair-inhibition increases the frequency and size of large deletions

**Figure 1.**
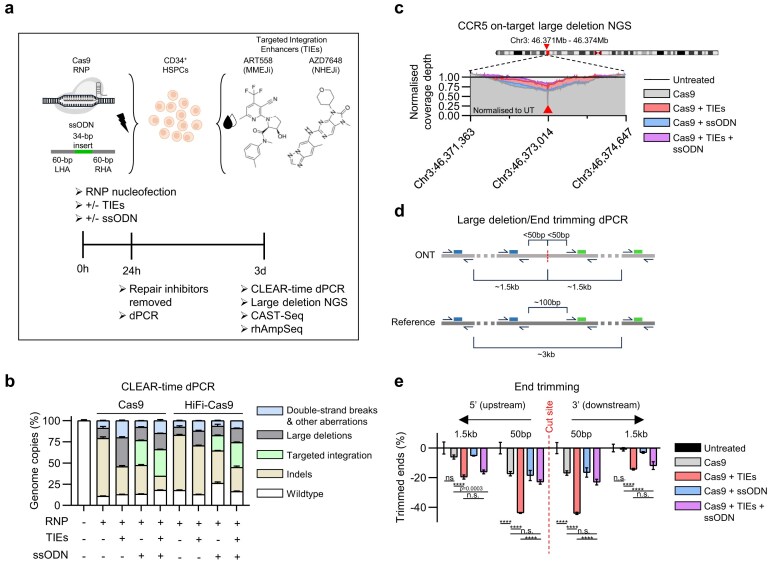
DSB repair inhibition increases the frequency and size of large deletions. (**a**) Schematic representation of Cas9 RNP editing strategy and experimental settings ± application of repair inhibitors and ssODN donor template. (**b**) CLEAR-Time dPCR characterization of on-target genome copies of CRISPR–Cas9-induced mutations with and without repair inhibitors and ssODN 3 days post-electroporation. Data shown as mean ± standard deviation. (**c**) Qualitative assessment of large deletions by NGS. Coverage-depth normalized to the highest coverage value in a 3-kb window and unedited cells. Red arrow-head points to nuclease cleavage site. *n* = 1 per treatment condition. (**d**) Schematic representation of dPCR strategy to quantify large deletions/end trimming proximal (50 bp) and distal (1.5 kbp) from the Cas9 cleavage site. ONT refers to on-target. (**e**) CLEAR-time dPCR analysis of genome copies characterized by end processing around the cleavage site. Red dotted line represents the Cas9 cleavage site. All data represents *n* = 3 technical replicates per condition unless stated otherwise. Data shown as mean ± s.d. Two-way ANOVA with Tukey post-hoc test. ****P < .0001. Data shown in Figs 1–4 and Supplementary Figs S1 and S4 are derived from the same set of samples.

We first explored whether repair inhibitors potentiated nuclease-induced mutations at the on-target locus. HSPCs were electroporated with a promiscuous sgRNA RNP complex targeting the C-C chemokine receptor type 5 (*CCR5*) gene, and an ssODN with homology to the on-target cleavage site and cultured with AZD7648 and ART558 DSB repair inhibitors (Fig. 1a and Supplementary Fig. S1a). Cleavage and Lesion Evaluation via Advanced Real-time dPCR (CLEAR-time), a new tool used to provide a comprehensive overview of editing outcomes, revealed in a recently published article that a significant fraction of loci ranging from 6% to 90% harbours unresolved DSBs or other aberrations and those will not be detected when performing NGS or long-read sequencing [21]. The HSPCs were cultured for 24 h with the TIEs after electroporation and we extracted the genomic DNA at day 3. As expected, TIEs increased the frequency of active DSBs and large deletions, though these were mitigated by the presence of an ssODN repair template donor (Fig. 1b). Interestingly, the ssODN insert sequence was found to be present in an anti-sense orientation, though this occurred at a low frequency and was not impacted by repair inhibition (Supplementary Fig. S1b). Previous studies have shown that DNA repair-inhibitors decrease the frequency of large deletions when assessed using NGS of amplicons [22]. Long-amplicon NGS coverage analysis, normalized over the unedited sample, appears to confirm this (Fig. 1c), however, this evaluation may be strongly biased by genomic fragmentation during DNA extraction, active DSBs and by a more efficient PCR amplification of small fragments [28]. For this reason, we leveraged CLEAR-time revealing an increased frequency and size of end processing at the 5′ and 3′ flanking regions of the on-target cleavage site in repair inhibited HSPCs, with up to 19.6 ± 1.2% loci having deletions ~1.5 kb from the cleavage site, from 6.1 ± 1.1% loci in Cas9 only edited cells (Fig. 1d and e). Again, the degree of end-processing was reduced in the presence of an ssODN donor template, or when a HiFi-Cas9 was utilized (Supplementary Fig. S1c and d), indicating a direct correlation between large deletions with recutting efficiency, and competition with other repair pathways. Taken together, these data show that large structural aberrations are exacerbated when DSBs are prohibited from repairing via end-joining mediated pathways but are mitigated when an exogenous donor-template is present.

### High-resolution CAST-Seq improves off-target discovery rate and validation

**Figure 2.**
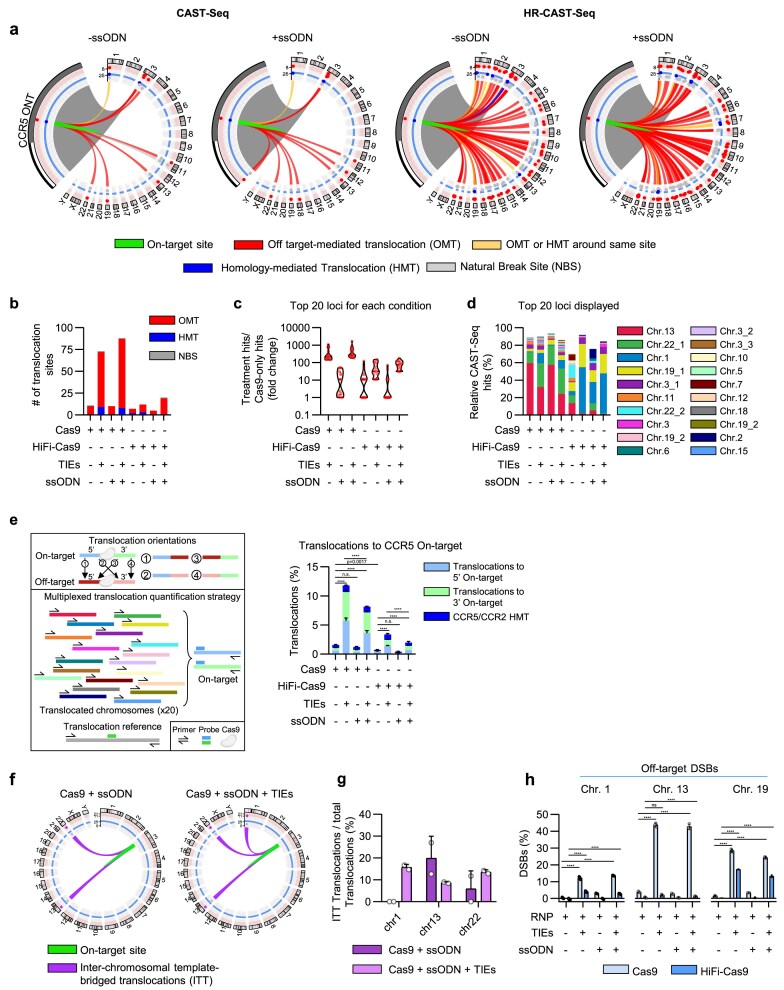
High-resolution CAST-Seq exposes the underlying nuclease off-targeting burden. (**a**) CAST-/HR-CAST-Seq circos plots 3 days post-editing in HSPCs at the *CCR5* gene locus, with and without repair inhibitors and ssODN donor template utilizing a promiscuous gRNA. Sites found in both replicates shown. Grey highlighted area shows a zoom of the site around the on-target site. (**b**) Total number of translocation partner sites detected with the *CCR5* on-target site. Sites found in both replicates shown. OMT = off-target-mediated translocation, HMT = homology-mediated translocation (cleavage at the on-target site only and repaired by HDR using a homologous sequence), NBS = natural break site. (**c**) The top 20 chromosomal aberration clusters for each sample condition were compared with the relative clusters on the Cas9 only sample in order to highlight the fold changes of the events (hits) measured in each cluster. Hits were averaged from *n* = 2 technical replicates per condition. (**d**) Relative translocation frequency among all translocations detected by CAST-Seq, top 20 translocations identified in Cas9-only edited HSPCs shown. (**e**) Schematic of possible translocation orientations and dPCR strategy to quantify the top 20 translocations identified by CAST-Seq. Four multiplex reactions were arranged per sample to evaluate the overall translocation burden with the on-target site. dPCR analysis of translocation frequency as a percentage of total alleles. Data shown as mean ± s.d. One-way ANOVA with Tukey post-hoc test. (**f**) Circos plot illustrating translocations between off-target loci and the on-target site with the donor template bridging them together. (**g**) Fraction of specific translocations that include the ssODN insert sequence over total. *n *= 2 technical replicates per condition. Data shown as mean ± s.d. (**h**) dPCR linkage analysis of DSB frequencies 24 h post-editing at off-target loci on chromosomes 1, 13, and 19. Data shown as mean ± s.d. Two-way ANOVA with Tukey post-hoc test. All data represent *n* = 3 technical replicates per condition unless stated otherwise. ****P < .0001. Data shown in Figs 1–4 and Supplementary Figs S1 and S4 are derived from the same set of samples.

CLEAR-time dPCR found that DSBs and other large structural aberrations, which include translocations, increased when Cas9-edited HSPCs were cultured with DSB-repair inhibitors. To explore this further, we performed CAST-Seq [17] on HSPCs from all treatment conditions. CAST-Seq revealed a 5–12-fold increase in off-target loci participating in translocations to the on-target site in repair-inhibited HSPCs, with NHEJ repair inhibition exerting the strongest effect on translocation formation (Supplementary Fig. S2a–d and Fig. 2a and b). This characteristic was therefore leveraged to create a ‘high-resolution’ version of the original CAST-Seq protocol (HR-CAST-Seq). Moreover, when assessing the diversity of translocations at each locus, we detected increases up to four orders of magnitude relative to Cas9 RNP-edited controls, highlighting the increased sensitivity of this approach (Fig. 2b–d). CAST-Seq shows consistent patterns in which the most common translocation sites exhibit higher frequencies with TIEs. However, stochastic fluctuations, primarily driven by replicate coverage and sequencing quality, result in ~3%–7% of translocation sites appearing more represented in samples without TIEs compared with TIE-treated samples.

To validate the translocation frequencies, we employed dPCR quantification using a reference sequence. Each off-target site exhibited up to four distinct translocation possibilities with alternative sequence partners (Fig. 2e). For detection of the top 20 off-target translocations involving the on-target site, we designed multiplex reactions combining forward or reverse primers specific to each site with either the forward or reverse primer for the on-target locus. This approach provided a robust quantitative assessment, corroborating the elevated levels of chromosomal aberrations observed across treatment conditions. It also verified the increased incidence of homology-mediated translocations at the CCR2 gene.

Notably, the presence of a donor template was associated with reduced translocation frequency for both Cas9 and its high-fidelity variant, irrespective of TIEs status, suggesting a protective role against chromosomal aberrations (Fig. 2e). The frequency trends were consistent with TQ-rhAmpSeq, dPCR, and even with CAST-seq whereas CAST-Seq reports that ssODN inclusion increases the nomination of off-target-mediated translocation sites in some conditions. This divergence suggests assay-specific differences in how off-target junctions are nominated in the presence of a donor template and warrants further investigation. In addition, since the ssODN was observed to integrate in an anti-sense orientation at the on-target site (Supplementary Fig. S1b), we further examined whether its integration also occurred between on-target and off-target translocated chromosome arms. Analysis of NGS data from CAST-Seq revealed the presence of interchromosomal template-bridged translocations (ITTs), with their frequency increasing when DSB repair was inhibited (Fig. 2f and g). To further investigate DSB persistence post-editing, we quantified DSBs at three prevalent off-target loci detected by CAST-Seq, 24 h after editing. All three off-target sites exhibited a substantial increase in the proportion of unresolved DSB loci in repair-inhibited cells, reaching up to 43.9 ± 0.9% DSBs at the chromosome 13 locus, which corresponded to the highest relative translocation frequency measured by CAST-Seq. As anticipated, the presence of an ssODN minimally affected Cas9 off-target cleavage efficiency (Fig. 2h). Although off-target activity cleavage activity was largely mitigated at chromosomes 1 and 13 with a HiFi-Cas9, they were still highly prevalent on chromosome 19 reflecting a sequence-specific selection of the high-fidelity protein. It is noteworthy that the CLEAR-time dPCR method, when coupled with repair inhibitors and HR-CAST-Seq, provides a sensitive and orthogonal confirmation of nominated off-target sites in relation to NGS deep sequencing (Fig. 2h and supplementary Fig. S2f and g). This is due to the higher detection of DSB percentages over indel percentages, in addition to reducing sequence-specific repair biases that might otherwise obscure cleavage activity.

Collectively, these results demonstrate that DSB repair inhibitors enhance the detection of novel translocation repair products and increase their frequency due to prolonged resolution of DSBs at on- and off-target sites.

### Promiscuous nuclease activity increases inter-off-target translocation frequency

**Figure 3.**
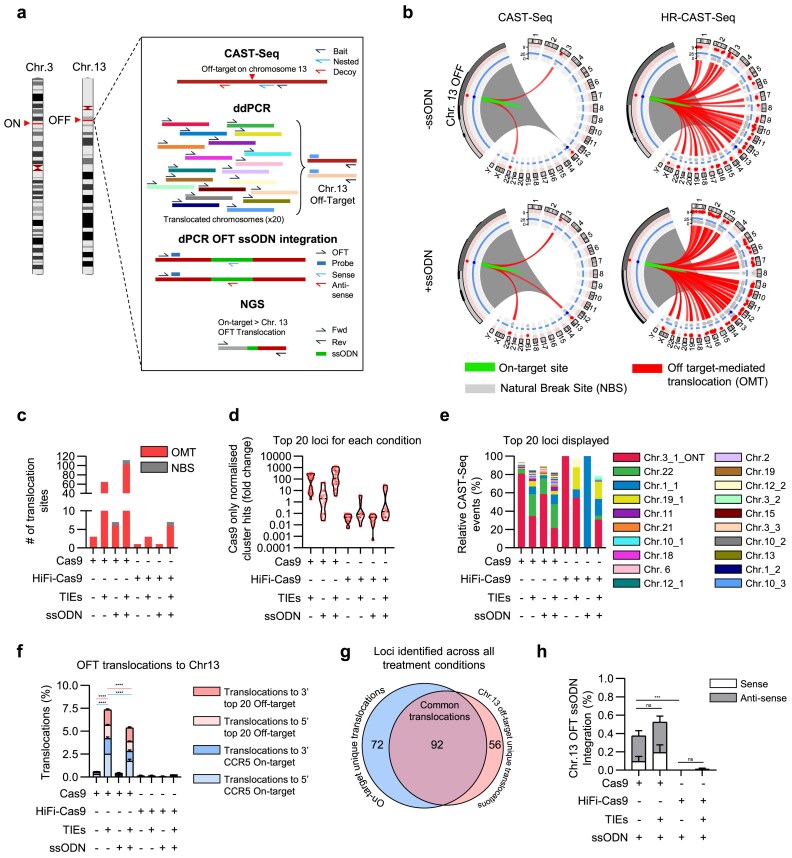
Inter-off-target transloc ation frequency. (**a**) Schematic illustrating experiments performed on the chromosome 13 off-target locus. (**b**) CAST-/HR-CAST-Seq circos plots 3 days post-editing in HSPCs at the chromosome 13 off-target locus, with and without DSB repair inhibitors and ssODN donor template. Sites found in both replicates shown. Grey highlighted area shows a zoom of the site around the on-target site. (**c**) Total number of loci translocating to the chromosome 13 off-target. Sites found in both replicates shown. OMT = off-target-mediated translocation, HMT = homology-mediated translocation, NBS = natural break site. (**d**) Fold change of the number of hits in each cluster relative to cluster hits in Cas9 only treatment condition. (**e**) Relative translocation frequency among all translocations detected by CAST-Seq, top 20 translocations shown. (**f**) dPCR analysis of translocation frequency as a percentage of total alleles between the *CCR5* on-target site and chromosome 13 off-target site (blue) and chromosome 13 off-target site and other off-target sites in the top 20 identified from the on-target CAST-Seq experiments. *n *= 3 technical replicates per condition. Data shown as mean ± standard deviation. One-way ANOVA with Tukey post-hoc test. ****P < .0001. (**g**) Total number of unique loci identified by CAST-Seq across all conditions and individual replicates at the *CCR5* on-target site (blue) and chromosome 13 off-target site (red). (**h**) dPCR analysis of integration of the ssODN into the chromosome 13 off-target site in both the sense and anti-sense orientation. *n *= 3 technical replicates per condition. Data shown as mean ± standard deviation. One-way ANOVA with Tukey post-hoc test. ***P = .0009. Data shown in Figs 1–4 and Supplementary Figs S1 and S4 are derived from the same set of samples.

HR-CAST-Seq enabled the identification and verification of nearly a hundred new off-target sites by utilizing a highly promiscuous sgRNA targeting the CCR5 locus (Fig. 2 and Supplementary Fig. S2). Many of these newly discovered off-targets appeared as single hit/replicate (hit = deduplicated reads by Sonic Abundance [35] representing the number of molecules present in the sample), approaching the detection limit defined by one significant hit in at least two replicates. Increasing the number of replicates expanded the list of identified loci while maintaining a low false-positive rate. To further investigate translocation events, we focused on one of the most frequently observed off-targets on chromosome 13 arising during CCR5 targeting (located on chromosome 3), in order to assess whether additional translocations were occurring between this site and other off-targets genome-wide, since cleavage at 24 h post-editing affected ~40% of the loci (Figs 2h and 3a).

With a primer strategy placed at the putative sgRNA binding site, identified on chromosome 13 off target, HR-CAST-Seq revealed over 100 translocation loci between the chromosome 13 off-target and other off-targets, a pattern substantially reduced when using a high-fidelity Cas9 variant (Fig. 3b–e). As expected, the majority of translocations occurred between chromosome 13 and the on-target site at *CCR5* (Fig. 3b and e, and Supplementary Fig. S3a). Similar to the on-target baited CAST-Seq, the cluster hits fold change significantly increased relative to the Cas9 only condition with off-target baited CAST-Seq, demonstrating that not only were the number of identified loci elevated but also the frequency at which this occurred (Fig. 3d and e). Highly multiplexed dPCR quantification corroborated these observations, revealing that ~3.1% of chromosome 13 loci translocated to selected off-target sites and ~4% to the on-target site. Inclusion of the ssODN reduced these events by ~1.2-fold (off-to-off combinations) and 1.5-fold (on-to-off combinations), indicating greater mitigation of translocation burden when the on-target site is involved (Fig. 3f). Across all treatment conditions, donors and CAST-Seq replicates, >200 loci were identified, underscoring a significant increase in detection sensitivity; notably, a substantial overlap was observed between loci nominated from analysis targeting both CCR5 and the chromosome 13 off-target site (Fig. 3g and Supplementary Fig. S3b).

NGS revealed ssODN donor template integrating into the chromosome 13 off-target locus, though dPCR confirmed this occurred at a relatively low frequency and was abrogated with a high-fidelity Cas9 variant (Fig. 3h). The ITTs between Chr. 3 and Chr. 13 showed a pattern of HDR with the *CCR5* ONT and always an ssODN-resected NHEJ or MMEJ mediated ligation with the cut site on Chr.13 off-target, in addition to novel donor template aberrations such as duplications (Supplementary Fig. S3c). The frequency of ITTs was not impacted by TIEs, but they did appear to impact end-processing prior to translocation with a greater diversity of junction sequences observed (Supplementary Fig. S3d).

Altogether, these data illustrate the profound impact of a promiscuous nuclease on genome stability. Under these circumstances, DSB repair inhibitors markedly increase aberration frequency, as every off-target site possesses the potential to translocate with other DSBs. Direct comparison with HiFi Cas9, both alone and in combination with TIEs, provides a more balanced assessment of DNA repair inhibition effects in respect to previous publications [26] and demonstrates that it is feasible to achieve minimal genomic instability if the editor is less promiscuous (Fig. 2b and 3c, and Supplementary Fig. S2e). Conversely, application of HR-CAST-Seq allows for comprehensive characterization and verification of off-target sites, and its deployment across multiple loci further validates the robustness of these findings.

### Off-targets validation and co-translocations quantification enhanced by rhAmpSeq adaptor randomization

It has been previously reported that canonical rhAmpSeq uses fixed P5/P7 ends on target-specific primers (Forward, P5; Reverse, P7) during amplicon sequencing [36, 37], which can result in some translocation events forming not compatible products (i.e. P5/P5; P7/P7) [34]. These events restrict the detection to only half of the possible unique translocation combinations between two cleaved sites.

**Figure 4.**
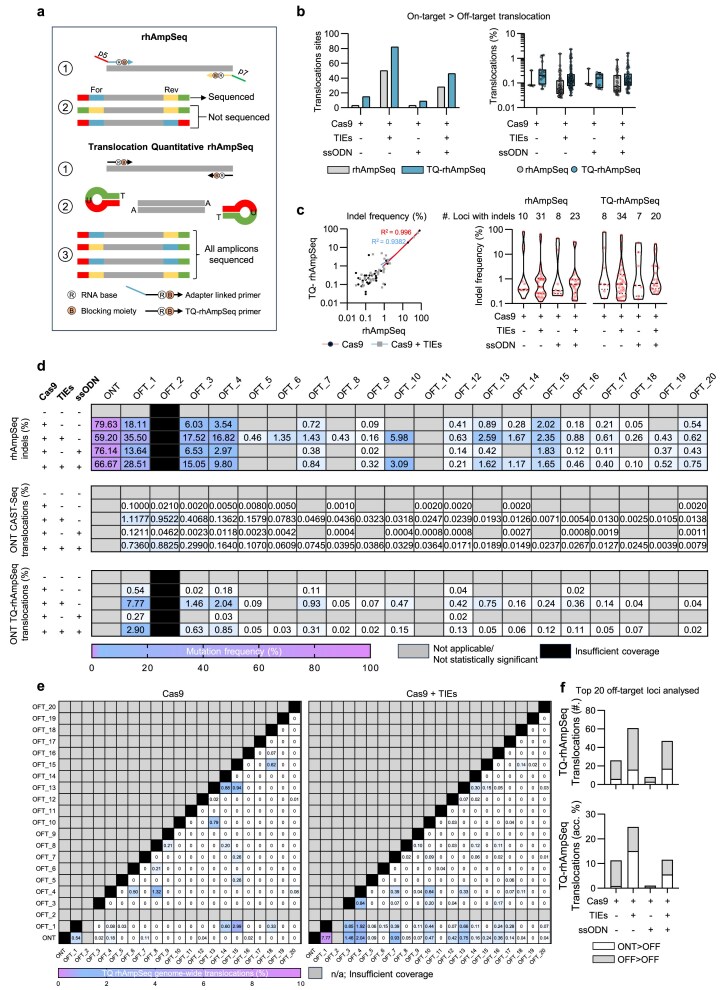
TQ-rhAmpSeq quantifies genome-wide off-target to off-target translocations. (**a**) Schematic illustrating TQ-rhAmpSeq adapter ligation strategy. (**b**) Comparison of 159 loci tested with rhAmpSeq and TQ-rhAmpSeq for the presence of translocation repair product (left) and cumulative translocation frequency across all loci (right). (**c**) Comparison of the indel frequency between rhAmpSeq and TQ-rhAmpSeq in Cas9 and Cas9 + TIEs treatment conditions (left) and total loci with an indel across all treatment conditions (right). (**d**) Tabular heatmaps displaying analysis of the top 20 loci for TQ-rhAmpSeq quantified indels (top), CAST-Seq translocations (middle), and TQ-rhAmpSeq quantified translocations (bottom). (**e**) Tabular heatmap of pairwise analysis for every potential translocation combination of the top 20 loci by TQ-rhAmpSeq in Cas9 and Cas9 + TIEs treatment conditions. (**f**) Bar graphs displaying total number of translocation combinations out of 190 possible different pairs (top) and cumulative translocation frequency (bottom) between on/off-target and off/off-target loci for the top 20 selected sites. Data shown in Figs 1–4 and Supplementary Figs S1 and S4 are derived from the same set of samples.

To address this, we implemented a modification synthesizing the target-specific primers without the adapter-amplification tails and attaching P7/P5 adapters post-PCR1 using a ligation approach enabling a compatible sequencing structure for all the molecules (Fig. 4a). We chose ~150 off-targets, based on the top off-target sites identified by HR-CAST-Seq, and investigated the performance of translocation quantification with and without this modification. We termed the modified translocation quantification version of rhAmpSeq using this approach ‘TQ-rhAmpSeq’.

As expected, TQ-rhAmpSeq recovered a greater number of loci participating in translocations compared with the classic rhAmpSeq primer design, increasing detected sites by up to ~5-fold. In parallel, the number of distinct translocations per locus increased, providing a broader view of the aberration burden present at the time of DNA collection (Fig. 4b). Notably, TQ-rhAmpSeq can be used to track the fate of both balanced and unbalanced translocations that generate acentric or dicentric chromosomes, which have been reported to be gradually lost over time in cells [17]. Conditions with NHEJ inhibition (NHEJi) exhibited a substantially higher translocation burden, consistent with CAST-Seq results.

Indel quantification rates were measured to test that editing quantification results were consistent between approaches. We found that performance was largely consistent between the classic rhAmpSeq strategy and TQ-rhAmpSeq both for conditions with (*R*^2 ^= 0.99) and without (*R*^2 ^= 0.93) TIEs. Noteworthy, NHEJ inhibition elevated indel frequencies across loci, increasing the number of statistically significant sites (Fig. 4c and Supplementary Fig. S4a).

After observing comparable indel results and increased translocation detection using TQ-rhAmpSeq, we compared translocation frequencies observed between orthogonal methods. The top significant translocations for TQ-rhAmpSeq and CAST-Seq largely correlated on rank-order of different off-target translocation and observed indel frequencies, though absolute frequencies differed between methods (Fig. 4d–e). Upon checking these frequencies, we saw relatively consistent cumulative ONT > OFF quantification frequencies between TQ-rhAmpSeq and dPCR further validating the approach, with dPCR quantifying between ~0.7% and 11% and TQ-rhAmpSeq between 0.3% and 15% (Figs 2f and 4f). These observation not only corroborated assessments from CAST-Seq but also demonstrated the sensitivity of the TQ-rhAmpSeq-based validation is high, approaching that of HR-CAST-Seq (Fig. 4d–f), and yielded accurate translocation quantification at the on-target locus comparable to multiplexed dPCR (Fig. 2f). A unique feature of TQ–rhAmpSeq, relative to CAST–seq and other existing methods, is its ability to quantify translocations among nominated off–targets at high sensitivity. In this study, we probed only 50 ng of genomic DNA for rhAmpSeq, equivalent to ~15 000 human haplotypes, yielding a theoretical limit of detection of 0.007%; the lowest observed value was 0.011% (translocation among OFT20 and OFT9), confirming the potential to interrogate even lower frequencies if required (Fig. 4a). As measurements approach these limits, it is important to balance input gDNA and per–site NGS coverage to minimize sensitivity bottlenecks.

### Assessing TIE–related chromosomal instability with precise nucleases

Since DSB repair inhibition mediated by AZD7648 potentiated chromosomal aberrations in gene-edited cells using a promiscuous sgRNA [26], we next investigated whether previously unreported aberrations could also be detected in cells edited with well-characterized sgRNAs. We selected *EMX1* for editing used in other studies [38] and therefore serves as a useful target for benchmarking HR-CAST-Seq. HSPCs were edited with an RNP complex targeting *EMX1* and cultured cells with and without DSB repair-inhibitors and analysed by CAST-Seq 3-days after editing. As anticipated, the HR-CAST-Seq identified more off-targets sites along with a characterization of larger deletions around the on-target site, due to the influence of the DSB repair inhibitors (Supplementary Fig. S5a–c). Additionally, we uncovered and verified different off-target sites by comparing our results with previously nominated sites from GUIDE-Seq analysis [38] (Supplementary Fig. S5d). Similarly, the number of translocations, large deletions, and small indels were confirmed with orthogonal methods such as dPCR (Supplementary Fig. S5e,), amplicon sequencing for indels (Supplementary Fig. S5f), and long-read amplicon sequencing for large deletions (Supplementary Fig. S5g). It is important to note that each assay inherently possesses biases in sensitivity and quantification, influenced by factors such as fragmented DNA, unresolved DSBs, or large deletions. Despite these limitations, the qualitative value of the data is enhanced by CLEAR-time, which provides quantitative harmonization across different assays especially in the presence of TIEs [21].

We then examined genomic integrity after editing with a GMP-like sgRNA targeting *WAS* based on its reported high-specificity and gene therapy application [39] to assess whether TIEs produce a higher rate of genomic aberrations under clinical–grade conditions.

**Figure 5.**
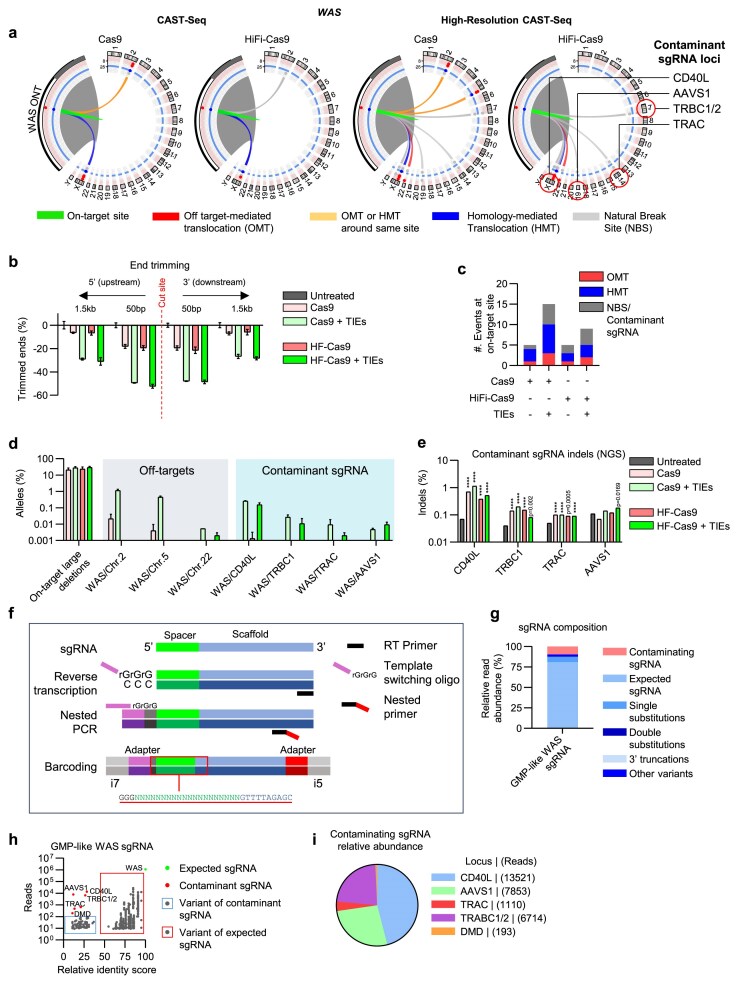
High-resolution CAST-seq unveils gRNA contaminants in GMP-like gRNA. (**a**) CAST-Seq circos plots 3 days post-editing in HSPCs at the *WAS* gene locus, with and without repair inhibitors utilizing a GMP-like sgRNA. Sites found in both replicates shown. Loci found translocated with on-target site due to gRNA contamination are listed in the last circos-plot. (**b**) CLEAR-time dPCR analysis of genome copies characterized by end processing around the cleavage site. Red dotted line represents the Cas9 cleavage site. All data represents *n* = 3 technical replicates per condition unless stated otherwise. Data shown as mean ± s.d. Two-way ANOVA with Tukey post-hoc test. ****P < .0001. (**c**) Total number of loci translocating to the *WAS* on-target site. Sites found in both replicates shown. OMT = off-target- mediated translocation, HMT = homology-mediated translocation (cleavage at the on-target site only and repaired by HDR using a homologous sequence), NBS = natural break site. (**d**) Bar-chart representing the percentage of alleles at the *WAS* editing site with large deletions, off-target translocations, and contaminant sgRNA editing mediated translocations. Fraction calculated based on starting DNA input. (**e**) NGS amplicon sequencing validation of indels at contaminating sgRNA editing site. Chi-square test over the relative untreated sample ****P < .0001. (**f**) Schematic representation of strategy to sequence sgRNAs. Red square highlights sequence of interest; the scaffold sequence in blue is utilized for bioinformatic positive selection. RT = reverse transcription. (**g**) Stacked-bar chart representing the composition of the sequenced sgRNA batch, comprised of the expected and contaminating sgRNA (together with mutant variants). (**h**) Scatter plot representing the relative identity score of the sequenced sgRNAs (compared to the expected sgRNA) and the number of NGS reads for each. (**i**) Pie chart depicting the relative abundance of the identified contaminating sgRNA sequences. Related data in Supplementary Figs S5h and i, and S6a–h.

As expected, the editing with HiFi-Cas9 yielded few translocation molecules, likely mediated by NBS and sequence homology, and CAST–seq detected no off–target-mediated translocations. In contrast, TIEs yielded a report identifying three off–target-mediated translocation sites with Cas9 and one with HiFi–Cas9, each close to the lower limit of detection (Fig. 5a–d and Supplementary Fig. S5h and i). These findings suggest that a precise nuclease combined with DNA-repair inhibitors can still deliver a stable genome with rare translocation events. CLEAR-time dPCR performed at 50 bp and 1.5 kb from the cut site, together with long-read amplicon sequencing, corroborated observations at CCR5, revealing an increased burden of end processing with TIEs (Fig. 5b and Supplementary Fig. S5i). Although large deletions increased under these conditions, they are typically considered lower risk in controlled settings and should be monitored; mechanistically, their predominant consequences often relate to gene dosage or loss-of-function at the target locus. In qualitative terms, this risk may not be comparable to that posed by translocations, which can form novel genomic junctions capable of ectopic promoter–gene fusions or enhancer hijacking with greater potential to dysregulate gene function. The modest increase in discovered off-target sites supports the use of AZD7648 in conjunction with well-designed and fully characterized sgRNAs for *ex vivo* protocols and potential clinical application.

### High-resolution CAST-Seq identifies sgRNA contaminants in GMP-like formulations

The sgRNA used to edit the *WAS* locus unexpectedly found translocations, which have not been previously described [39]. Upon deeper investigation of the translocation products, we found the loci are commonly edited for targeted gene therapies in HSPCs and T Cells [11, 40–42], potentially due to editing from unintentional carry over from sgRNA manufacture (Fig. 5a–e and Supplementary Fig. S6a). The translocations were rare and only detected using HR-CAST-Seq but were observed in independent editing experiments using also a high-fidelity Cas9 lending further support to sgRNA manufacturing contamination rather than off-target Cas9 activity from sgRNA mismatches (Fig. 5d). Non-specific editing was confirmed by NGS by quantifying indels at these loci, with indels at the *CD40L* locus found to be the highest among all contaminant loci reaching 1.14% in one instance (Fig. 5e). This mirrored the higher prevalence of translocations between *WAS* and *CD40L* using HR-CAST-Seq; an observation that was confirmed by direct PCR for the *CD40L*/*WAS* translocation (Supplementary Fig. S6h and i).

To confirm the presence of unintentional sgRNA carry-over, we developed a streamlined sgRNA processing and sequencing pipeline (sgRNA contamination and read integrity analysis by enriched NGS; SCRIBE-Seq) (Fig. 5f). sgRNA was reverse transcribed with a primer on the scaffold and in the presence of a TSO encoding an adapter for NGS barcoding followed by a nested PCR using a primer also encoding an NGS adapter. SCRIBE-Seq found the majority of sgRNAs were the expected sequence but was mixed with variants of the expected sgRNAs including substitutions and truncations (Fig. 5g and h). Among the expected sgRNA variants, we indeed identified sgRNA sequences that mapped to the translocated loci found by HR-CAST-Seq (also with variants), in addition to an apparent disease specific sgRNA targeting exon 51 in *DMD* (Fig. 5h and Supplementary Fig. S6b). A contaminating sgRNA targeting *CD40L* was the most prevalent, which explains the higher number of translocations and indels compared to the other contaminants (Fig. 5i). sgRNA contaminants were not exclusive to GMP-like formulations, as we also identified them in smaller scale research grade formulations (Supplementary Fig. S6c–f). Translocations arising from contaminant sgRNAs were validated by direct PCR amplification of sgRNA cDNA (Supplementary Fig. S6g), and the WAS/CD40L translocation were additionally detected in separate editing experiments utilizing GMP-like sgRNA (Supplementary Fig. S6i). Notably, neither our group nor other teams within the laboratory had previously targeted these loci, making the possibility of end-user cross-contamination highly unlikely.

Collectively, these data show that HR-CAST-Seq greatly improves the sensitivity translocation detection to the degree of identifying multiple low-yield, yet biologically active, contaminating sgRNA species.

## Discussion

The rapid progress of genetic therapies continues to catalyse the discovery of new enzymes and editing modalities, with implications that extend beyond clinical translation into fundamental biology and technology development. Small–molecule modulation of DNA repair is being explored to attenuate host responses to donor DNA and/or to increase HDR efficiency [10]. In our previous work, we used the DNA–PKcs inhibitor AZD7648 to resolve precise NHEJ repair outcomes, delineating cut–repair cycles elicited by designer nucleases and quantifying editing mutational rates without known assay bias [21].

In this study, we elucidated the genome destabilization associated with DNA repair inhibitor interventions and provide a balanced evaluation of their effects. We leverage these mechanistic properties to develop a more sensitive toolkit for assessing genome stability and identify new safety concerns related to sgRNA cross-contamination in research- and GMP-like batches. Specifically, we introduce more sensitive, orthogonal approaches for off–target nomination and validation: high–resolution CAST–Seq and translocation–quantitative rhAmpSeq.

First, CLEAR-time dPCR revealed the true burden of large deletions following AZD7648, overcoming biases that affect long-amplicon sequencing [28]. Loss and/or transposition of genomic segments and unresolved DSBs introduce inherent biases in quantifying Cas9-induced large deletions, for example, due to the loss of a primer binding sites preventing efficient PCR amplification. We show that the size and frequency of Cas9-induced large deletions are potentiated by DSB-repair inhibitors through different methods, and while the long-amplicon sequencing provided solid signal, its quantitative readouts diverged from dPCR, consistent with the amplification biases foreseen. Previous articles reported an exacerbated effect due to a suboptimal experimental design that subtracted different genome copies from the evaluations [22, 26]. In particular, genomic DNA was frequently harvested 1–3 days post-treatment without removing the NHEJ inhibitor, a window in which a substantial fraction of genome copies can remain unrepaired (on the order of 10%–90% [21]). Additionally, long-read sequencing performed after long-range PCR can stochastically over-represent shorter amplicons, shifting estimates toward a higher apparent burden of large deletions [28]. This amplification-length bias was the main reason we refrained using long-amplicon sequencing for quantitative inference in this study.

It is therefore important to exercise caution when interpreting such data, as failing to take these biases into account can overestimate large–deletion prevalence while simultaneously underrepresenting other aberrations. By contrast, CLEAR-time dPCR directly measures locus-specific copy number and deletion burden without relying on intact primer sites or sequence, providing more robust quantification under DSB-repair inhibition.

The true extent of Cas9 cleavage activity is obscured by the relative precision and speed of the NHEJ repair pathway resulting in a scarless repair product [43, 21]. Such repair evades detection by techniques such as GUIDE-Seq or CAST-Seq, which identify Cas9 activity by relying on the integration of oligonucleotides into off-target DSBs or the formation of structural variations, respectively. By extending the time Cas9-induced DSBs remain unresolved with DNA repair inhibitors, we found an increased likelihood of genomic translocation thus demonstrating that Cas9 activity at off-target loci is more prevalent than previously assumed. Accumulation of genome-wide DSBs via off-target Cas9 cleavage activity and extension of their resolution thereby maximizes the likelihood of a translocation in agreement with previous findings [23]. The frequency of translocation formation in this context reflects that seen by class-switching recombination, which is largely dependent on the rate of DSB generation and their persistence [44].

Then, we quantified the impact of donor templates on structural outcomes, finding that donor inclusion consistently mitigates translocation frequency but does not invariably reduce large deletions that extend beyond the homology arm length. Although translocations decreased with a donor, we observed rare events in which donor sequence was interposed between the on–target locus and an off–target junction. These observations suggest previously unrecognized overlap between ostensibly independent repair pathways [45] and reveal a novel aberration that introduces additional genotoxic risk, both of which merit further investigation.

By performing HR-CAST-Seq on the most prominent off-target as bait (chromosome 13) of a *CCR5* promiscuous sgRNA, we demonstrated that translocations occur at a high frequency between off-targets, and in doing so, we further identified loci missed with the on-target HR-CAST-Seq. Additionally, we unsurprisingly show minimal impact on translocations between off-targets with the inclusion of a donor template due to a lack of sequence homology with off-target loci. Together, this demonstrates the increased sensitivity of HR-CAST-Seq but also the need to be wary of ‘background’ activity regarding Cas9 cleavage and cellular repair, which is often unwittingly missed in genotoxic screening analysis.

Using an empirically defined list of targets derived from HR-CAST-Seq, and accounting for the occurrence of multiple translocation orientations, we elucidated the occurrence of translocations between off-target loci. We made a subtle yet effective amendment to rhAmpSeq, a technique already implemented as an orthogonal validation method for off-target screening [46–48]. With this modification, relative indel quantification remained concordant with the classic rhAmpSeq technique with the additional benefit of quantifying translocations between off-target sites building upon previous recommendations [34]. Thus, within a single assay, we provide two orthogonal modalities for off-target validation: indel burden and the presence of translocations involving the intended and/or off-target loci. Notably, DNA-PKcs inhibition further increased assay sensitivity by more than an order of magnitude.

Using HR-CAST-Seq, we confirmed a minimal translocation burden arising from off-target nuclease activity with previously described ‘precise’ sgRNAs. This supports the view that, in *ex vivo* applications, DNA–PKcs inhibition to enhance HDR may be compatible with highly precise nucleases when coupled with stringent guide selection, manufacturing QC (to exclude contaminant guides), and comprehensive genomic safety profiling. Nonetheless, we observe that on–target large deletions persist and can increase under TIEs, underscoring the need to reliably quantify both translocations and deletions in any clinical program. However, when increasing CAST–Seq sensitivity under DNA–PKcs inhibition, we detected unintended contaminating sgRNA molecules within the GMP-like formulation. Arguably, target-specific contaminating sgRNAs likely pose greater genotoxic risk than stochastic off-target-driven translocations because deliberately designed guides are more prone to engage biologically relevant loci. Although this demonstrates the improved detection sensitivity of HR-CAST-Seq, it critically demonstrates the necessity of more stringent quality control of GMP-like batches. To complement HR-CAST-Seq, we optimized sgRNA NGS using semi-nested, TSO-based RT-PCR, revealing additional contaminants, including guides designed for specific *DMD* mutations and sequence variants of the intended guide. Recent comparative analyses across vendors suggest that such events may be more frequent than anticipated in research-grade sgRNAs, reinforcing the need for revising production standards [49]. These observations warrant reassessment of off-target nomination filters and highlight manufacturing guide-purity challenges, underscoring the need for sustainable, cost-effective production.

In conclusion, this study emphasizes the central role of NHEJ pathways in maintaining genomic stability. By leveraging these repair properties, we developed HR-CAST-Seq and TQ-rhAmpSeq, providing a more sensitive and orthogonal view of genome-wide integrity while reducing false-positive off-target nomination in the context of designer nucleases. Clearer, orthogonally validated off-targets are likely to strengthen data sets for model training, thereby improving the performance and true discovery rate of *in silico* prediction tools. Moreover, we support the judicious use of DSB-repair inhibitors to improve therapeutically relevant outcomes, provided the nucleases are rigorously characterized.

## Supplementary Material

gkag318_Supplemental_Files

## Data Availability

Source data are provided with this paper. NGS fastq data are available by the NCBI SRA database PRJNA1380439 http://www.ncbi.nlm.nih.gov/bioproject/1380439.
