# Supplementary material for: DNA-PKcs inhibitor AZD7648 reveals sgRNA cross-contaminants and enhanced sensitivity of genome engineering off-target activity in HSPCs
Source: Nucleic Acids Res. 2026 Apr 20;54(7):gkag318. doi: 10.1093/nar/gkag318 (PMC13092968; doi:10.1093/nar/gkag318)
Supplement: gkag318_Supplemental_Files [file gkag318_supplemental_files.zip › Supplementary figures.pdf]

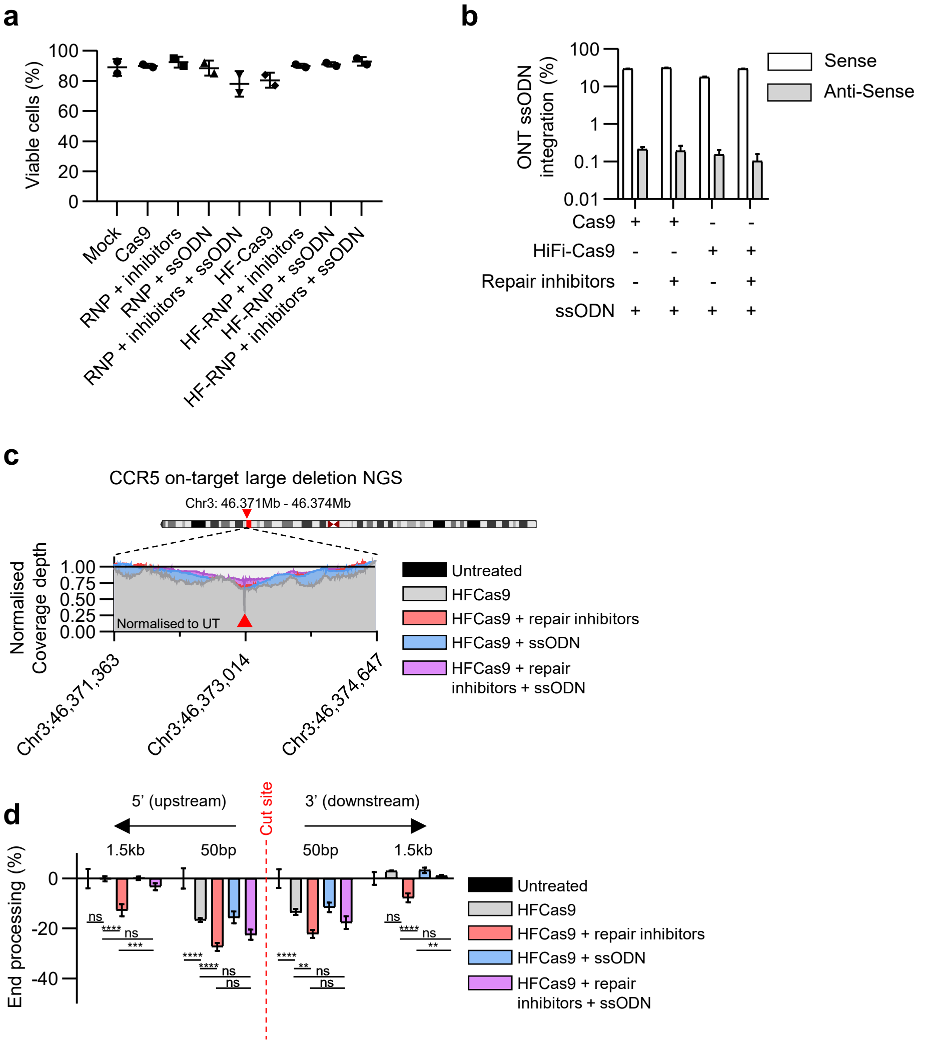

Supplementary Figure 1 | DSB repair inhibition increases the frequency and size of large deletions

(**a**) Cell viability of each condition at the time of collection for analysis (**b**) Orientation of ssODN insert at the on-target site by ddPCR. (**c**) Qualitative assessment of large deletions by NGS of HSPCs edited with a high-fidelity Cas9 variant. Coverage-depth normalised to the highest coverage value in a 3-kb window and unedited cells. Red arrow-head points to nuclease cleavage site. (Related to Fig. 1c). (**d**) dPCR analysis of genome copies characterised by end processing around the cleavage site of HSPCs edited with HiFi-Cas9. n = 3 technical replicates per condition. Data shown as mean ± standard deviation. Two-way ANOVA with Tukey post-hoc test. P <0.0001. Red dotted line represents the Cas9 cleavage site. (Related to Fig. 1e). Data shown in Figure 1-4, supplementary Figure1,4 are derived from the same set of samples.

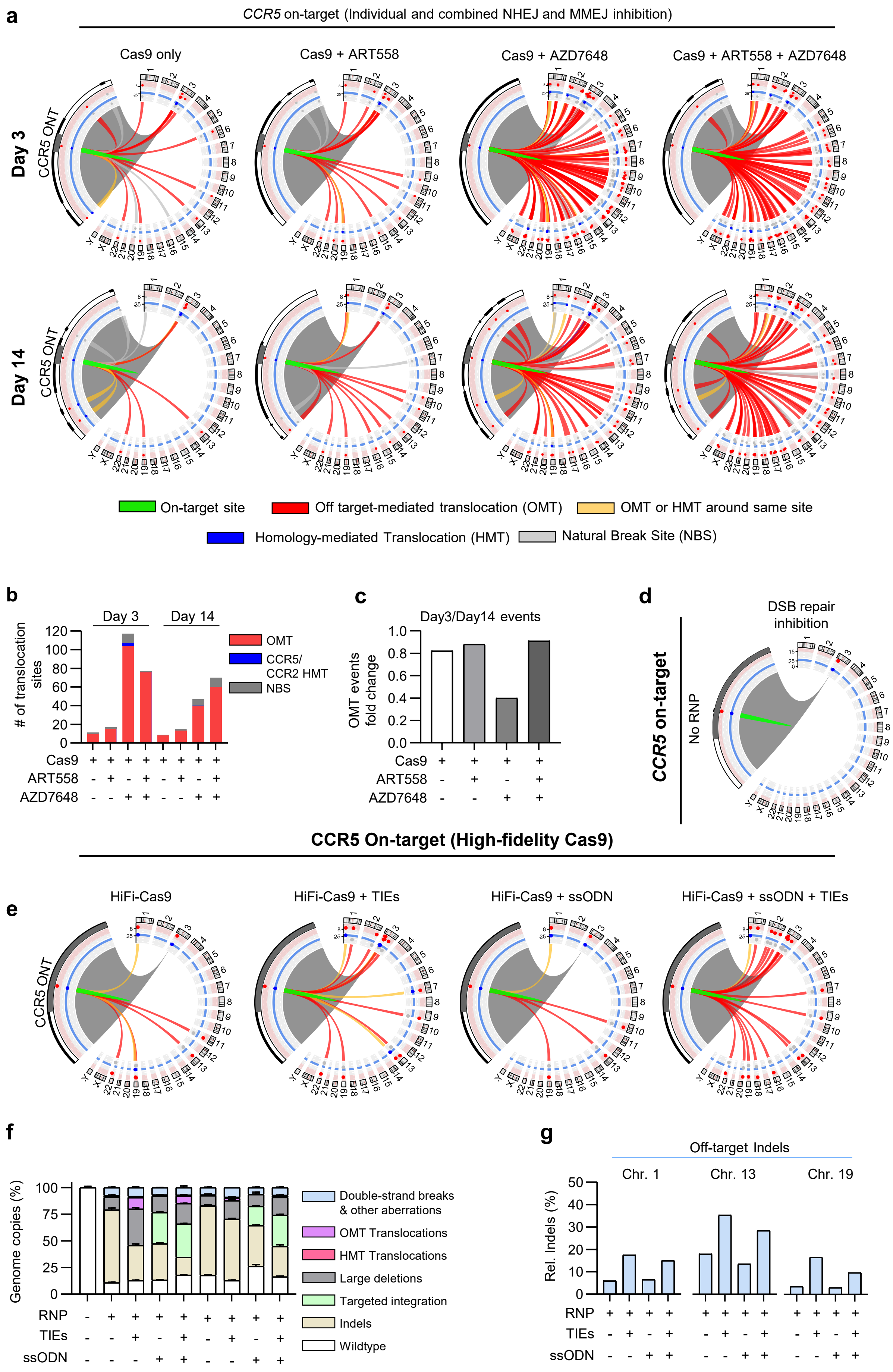
a
CCR5 on-target (Individual and combined NHEJ and MMEJ inhibition)
Cas9 only
Cas9 + ART558
Cas9 + AZD7648
Cas9 + ART558 + AZD7648
Day 3
CCR5 ONT
Day 14
CCR5 ONT
On-target site
Off target-mediated translocation (OMT)
OMT or HMT around same site
Homology-mediated Translocation (HMT)
Natural Break Site (NBS)
b
Day 3
Day 14
# of translocation sites
OMT
CCR5/ CCR2 HMT
NBS
Cas9
ART558
AZD7648
c
Day3/Day14 events
OMT events fold change
Cas9
ART558
AZD7648
d
DSB repair inhibition
CCR5 on-target
No RNP
CCR5 On-target (High-fidelity Cas9)
e
HiFi-Cas9
HiFi-Cas9 + TIEs
HiFi-Cas9 + ssODN
HiFi-Cas9 + ssODN + TIEs
CCR5 ONT
f
Genome copies (%)
Double-strand breaks & other aberrations
OMT Translocations
HMT Translocations
Large deletions
Targeted integration
Indels
Wildtype
RNP
TIEs
ssODN
g
Off-target Indels
Chr. 1
Chr. 13
Chr. 19
Rel. Indels (%)
RNP
TIEs
ssODN

Supplementary Figure 2 | High-Resolution CAST-Seq reveals increased Cas9 off-target cleavage activity

(**a**) CAST-Seq circos plots 3- and 14-days post-editing in HSPCs at the *CCR5* gene locus, with individual or combined DSB repair inhibitors utilising a promiscuous gRNA. Sites found in both replicates shown. (**b**) Total number of loci translocating to the *CCR5* on-target site. Sites found in both replicates shown. OMT= off-target mediated translocation, HMT= Homology mediated translocation (cleavage at the on-target site only and repaired by homology-directed repair using a homologous sequence), NBS= natural break site. (**c**) Relative change of the number of loci across different treatments between the day 3 and day 14 timepoints. (**d**) CAST-Seq circos plot of DSB-repair inhibited only cells with no editing. (**e**) CAST-/HR-CAST-Seq circos plots of *CCR5* edited HSPCs using a high-fidelity Cas9 variant 3 days post editing. (Related to Fig. 2a). (**f**) Translocation appended CLEAR-time stacked bar chart from Fig. 1b. (**g**) Next generation sequencing for indels at known off-target loci 3 days post-editing with Cas9.

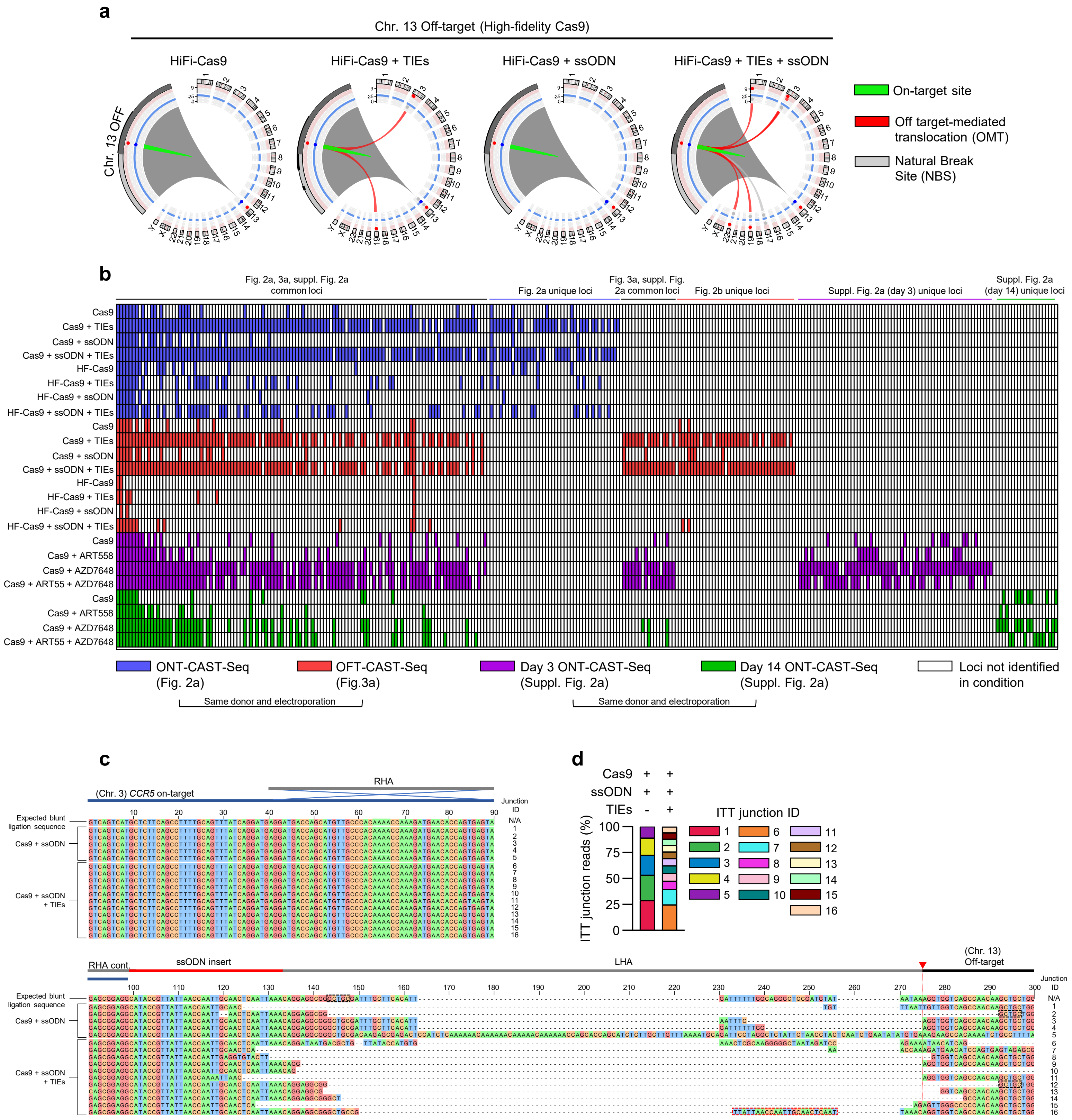

Supplementary Figure 3 | High off-target Cas9 activity increases inter-off-target translocation frequency

(**a**) CAST-/HR-CAST-Seq circos plots 3 days post-editing in HSPCs at the chromosome 13 off-target locus, with and without DSB repair inhibitors and ssODN donor template utilising HiFi-Cas9. Sites found in both replicates shown. (**b**) Heatmap representing the off-target sites overlap among different conditions, donors and days after editing the CCR5 site in HSPCs. (**c**) Multiple Alignment using Fast Fourier Transform (MAFFT) alignment of directly sequenced ITT between the chromosome 3 on-target and chromosome 13 off-target. Alignment performed using expected sequence if donor template bluntly ligates between the two loci as a reference (top sequence), with annotation of this expected sequence shown above. Black dotted box = microhomology, red dotted box= donor template, red arrowhead/dotted line= cleavage site at chromosome 13 off-target. (**d**) Stacked bar chart of relative distribution frequency of ITT junction reads.

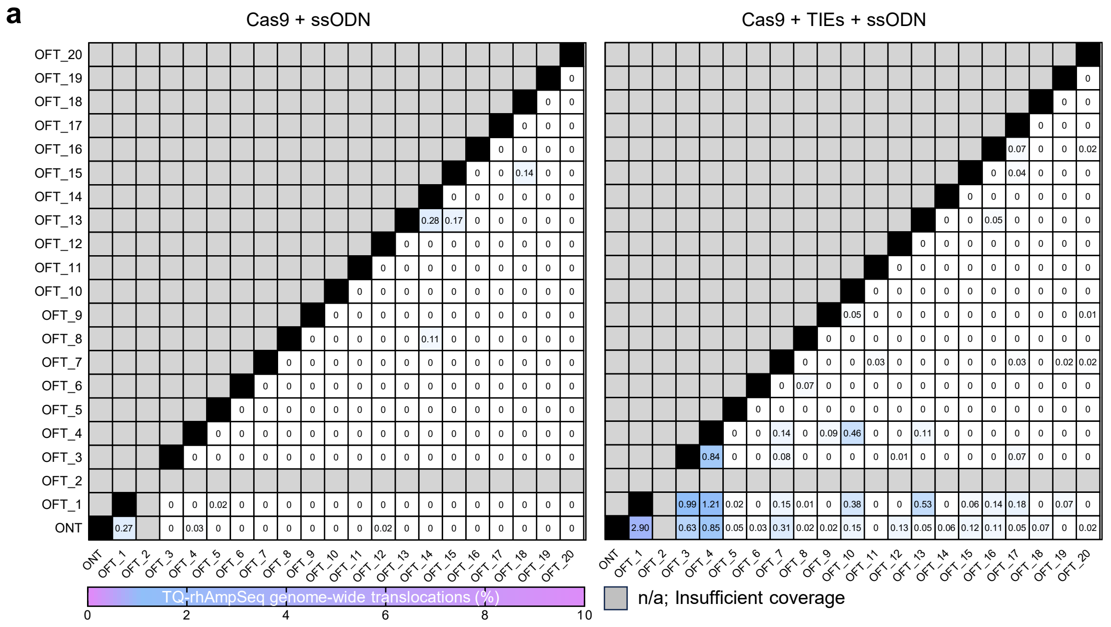

Supplementary Figure 4 | TQ-rhAmpSeq quantifies genome-wide off-target to off-target translocations

(**a**) Tabular heatmap of pairwise analysis for every potential translocation combination of the top 20 loci by TQ-rhAmpSeq in Cas9 + ssODN and Cas9 + TIEs + ssODN treatment conditions. Data shown in Figure 1-4, supplementary Figure1,4 are derived from the same set of samples.

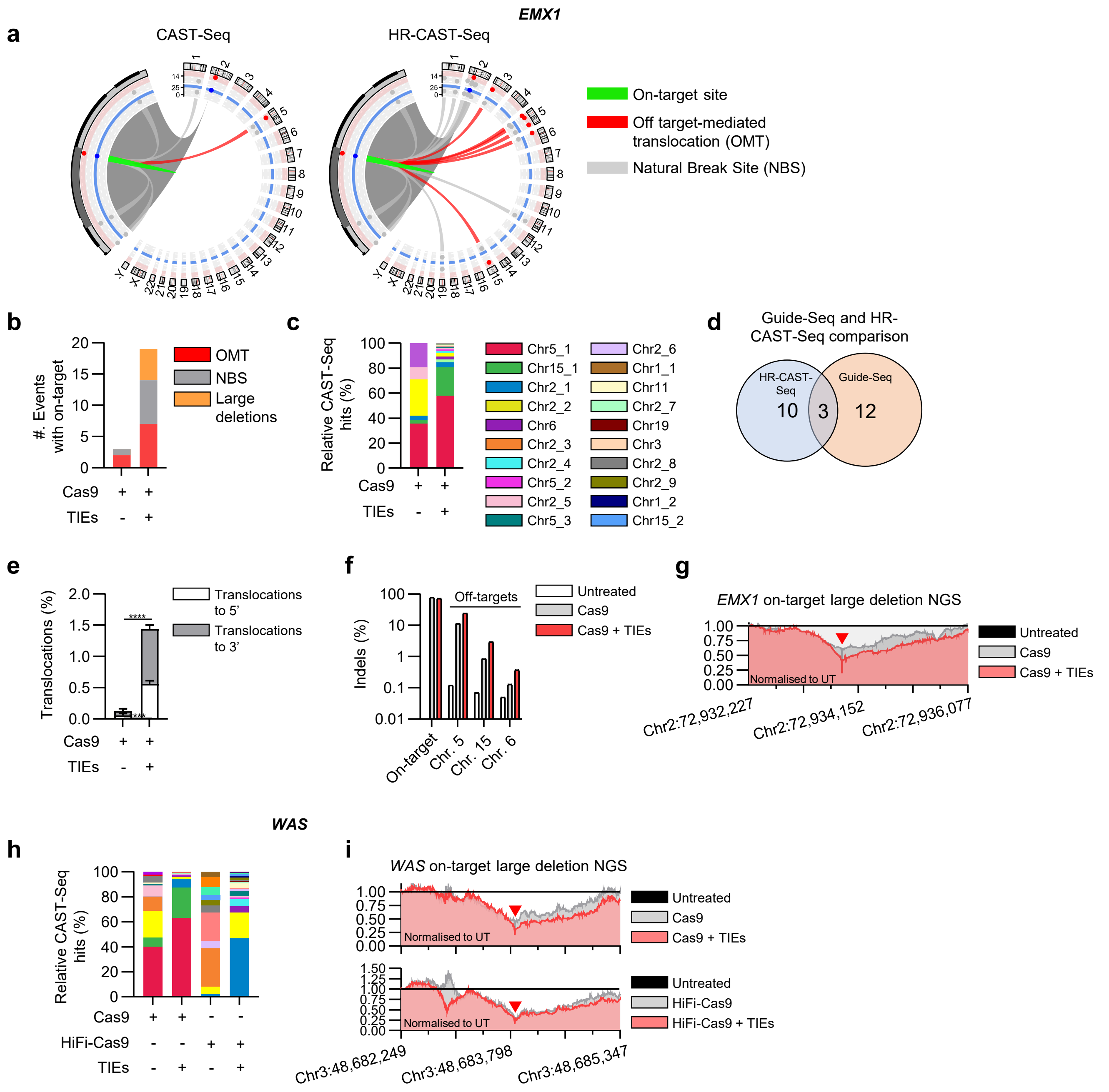

Supplementary Figure 5 | Repair profile of moderate/non-promiscuous sgRNAs
(**a**) CAST-/HR CAST-Seq circos plots 3 days post-editing in HSPCs at the *EMX1* locus, with and without repair inhibitors. (**b**) Total number of events (large deletions, translocations etc.) with the *EMX1* on-target site. Sites found in both replicates shown. OMT = off-target mediated translocation, NBS = natural break site. (**c**) Relative translocation frequency amongst all translocations detected by CAST-Seq, top 20 translocations identified in Cas9-only edited HSPCs shown. (**d**) Venn diagram illustrating identified off-target loci between HR-CAST-Seq in HSPC and GUIDE-Seq loci in HEK293T cells (from Tsai at al.. Nat Biotechnol. 2015). (**e**) Highly multiplexed dPCR for translocations shown as a percentage of total alleles. Data shown as mean ± standard deviation. one-way ANOVA with Tukey post-hoc test. ****p <0.0001. (**f**) NGS amplicon sequencing for indels of identified off-targets. (**g**) Qualitative assessment of large deletions by NGS of Cas9 edited HSPCs targeting *EMX1*. Coverage-depth normalised to the highest coverage value in a 3-kb window and unedited cells. Red arrow-head points to nuclease cleavage site. (**h**) Relative translocation frequencies in WAS edited HSPC. (**i**) Qualitative assessment of large deletions by NGS of Cas9 edited HSPCs targeting *WAS*. Coverage-depth normalised to the highest coverage value in a 3-kb window and unedited cells. Red arrow-head points to nuclease cleavage site.

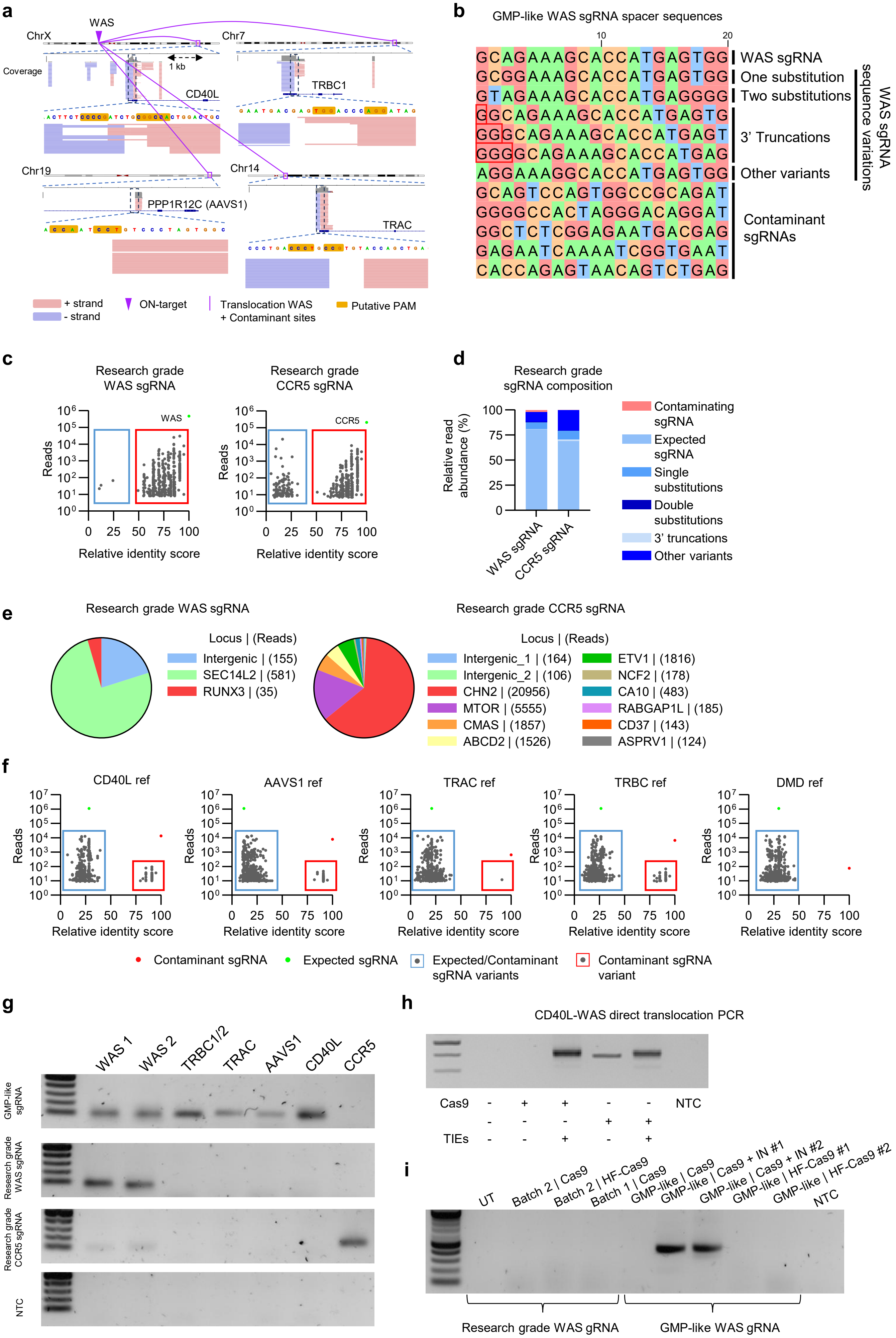
a
WAS
ChrX
Chr7
Coverage
1 kb
CD40L
TRBC1
Chr19
Chr14
PPP1R12C (AAVS1)
TRAC
+ strand
- strand
ON-target
Translocation WAS + Contaminant sites
Putative PAM
b
GMP-like WAS sgRNA spacer sequences
GCAGAAAGCACCATGAGTGG WAS sgRNA
GCGGAAAGCACCATGAGTGG One substitution
GTAGAAAGCACCATGAGGGG Two substitutions
GGCAGAAAGCACCATGAGTG
GGGCAGAAAGCACCATGAGT
GGGGCAGAAAGCACCATGAG
3' Truncations
AGGAAAGGCACCATGAGTGG Other variants
WAS sgRNA sequence variations
GCAGTCCAGTGGCCGCAGAT
GGGGCCACTAGGGACAGGAT
GGCTCTCGGAGAATGACGAG
GAGAATCAAAATCGGTGAAT
CACCAGAGTAACAGTCTGAG
Contaminant sgRNAs
c
Research grade WAS sgRNA
Research grade CCR5 sgRNA
Reads
Relative identity score
d
Research grade sgRNA composition
Relative read abundance (%)
WAS sgRNA
CCR5 sgRNA
Contaminating sgRNA
Expected sgRNA
Single substitutions
Double substitutions
3' truncations
Other variants
e
Research grade WAS sgRNA
Locus | (Reads)
Intergenic | (155)
SEC14L2 | (581)
RUNX3 | (35)
Research grade CCR5 sgRNA
Intergenic_1 | (164)
Intergenic_2 | (106)
CHN2 | (20956)
MTOR | (5555)
CMAS | (1857)
ABCD2 | (1526)
ETV1 | (1816)
NCF2 | (178)
CA10 | (483)
RABGAP1L | (185)
CD37 | (143)
ASPRV1 | (124)
f
CD40L ref
AAVS1 ref
TRAC ref
TRBC ref
DMD ref
Contaminant sgRNA
Expected sgRNA
Expected/Contaminant sgRNA variants
Contaminant sgRNA variant
g
WAS 1
WAS 2
TRBC1/2
TRAC
AAVS1
CD40L
CCR5
GMP-like sgRNA
Research grade WAS sgRNA
Research grade CCR5 sgRNA
NTC
h
CD40L-WAS direct translocation PCR
Cas9
TIEs
NTC
i
UT
Batch 2 | Cas9
Batch 2 | HF-Cas9
Batch 1 | Cas9
GMP-like | Cas9
GMP-like | Cas9 + IN #1
GMP-like | Cas9 + IN #2
GMP-like | HF-Cas9 #1
GMP-like | HF-Cas9 #2
NTC
Research grade WAS gRNA
GMP-like WAS gRNA

Supplementary Figure 6 | High resolution CAST-seq unveils sgRNA contaminants in GMP-like sgRNA formulation

(**a**) IGV visualization of translocation sites between WAS and contaminant sgRNA editing loci. (**b**) Representative sgRNA sequences identified with SCRIBE-Seq. Red boxes around 3' truncated sequences highlight stretches of Gs added during reverse transcription. (**c**) SCRIBE-Seq reads/relative identity score analysis of research grade sgRNAs. (**d**) sgRNA composition of research grade sgRNAs (**e**) Relative abundance of sgRNA contaminants. (**f**) NGS reads/relative identity score of sgRNA contaminants in the GMP-like *WAS* sgRNA preparation using the contamination sequence as a reference for identity scoring. (**g**) Direct PCR of contaminant sgRNAs using cDNA from reverse transcribed sgRNA. (**h**) Direct PCR of CD40L/WAS translocation validating HR-CAST-Seq observations resolved on 2% agarose gel; related with Fig. 5 and Supplementary Fig. 5i-k and derived from the same set of samples. (**i**) Direct PCR of the *CD40L/WAS* translocation with DNA from previous editing experiments utilising research or GMP-like WAS sgRNAs.
